# Bridge Damage Identification Using Vehicle Bump Based on Additional Virtual Masses

**DOI:** 10.3390/s20020394

**Published:** 2020-01-10

**Authors:** Qingxia Zhang, Jilin Hou, Łukasz Jankowski

**Affiliations:** 1 School of Civil Engineering, Dalian Minzu University, Dalian 116600, China; 2 Department of Civil Engineering & State Key Laboratory of Coastal and Offshore Engineering, Dalian University of Technology, Dalian 116023, China; 3 Institute of Fundamental Technological Research, Polish Academy of Sciences, 02-106 Warsaw, Poland; ljank@ippt.pan.pl

**Keywords:** structural health monitoring, damage identification, vehicle bump, additional virtual mass, bridge

## Abstract

Structural damage identification plays an important role in providing effective evidence for the health monitoring of bridges in service. Due to the limitations of measurement points and lack of valid structural response data, the accurate identification of structural damage, especially for large-scale structures, remains difficult. Based on additional virtual mass, this paper presents a damage identification method for bridges using a vehicle bump as the excitation. First, general equations of virtual modifications, including virtual mass, stiffness, and damping, are derived. A theoretical method for damage identification, which is based on additional virtual mass, is formulated. The vehicle bump is analyzed, and the bump-induced excitation is estimated via a detailed analysis in four periods: separation, free-fall, contact, and coupled vibrations. The precise estimation of bump-induced excitation is then applied to a bridge. This allows the additional virtual mass method to be used, which requires knowledge of the excitations and acceleration responses in order to construct the frequency responses of a virtual structure with an additional virtual mass. Via this method, a virtual mass with substantially more weight than a typical vehicle is added to the bridge, which provides a sufficient amount of modal information for accurate damage identification while avoiding the bridge overloading problem. A numerical example of a two-span continuous beam is used to verify the proposed method, where the damage can be identified even with 15% Gaussian random noise pollution using a 1-degree of freedom (DOF) car model and 4-DOF model.

## 1. Introduction

Bridges are key components of transportation infrastructure. In recent decades, due to environmental effects, the ageing process, and increased traffic loading [1], the demand of bridge condition monitoring to maintain the service safety of bridges is increasing [2]. Structural health monitoring (SHM) [3,4,5,6,7,8] has been extensively employed in practical civil engineering projects, especially for large-scale structures such as bridges [9,10]. Structural damage identification [11,12,13,14,15] has an important role in providing effective evidence for bridge maintenance and assessment. 

In structural dynamics, if the system parameters and its excitations are known, the calculation of the corresponding structural response is a direct problem. The identification of system parameters or excitations using the known response constitutes an inverse problem, and damage identification is thus an inverse problem. Structural dynamic characteristics are often used to detect damage by assessing their changes between an intact structure and a damaged structure. Structural modal parameters, such as natural frequencies, mode shapes, modal mass and stiffness, are popular as dynamic indices for damage identification [16,17,18,19], and are easily obtained due to the rapid development of experiment modal analysis techniques. For example, Wang et al. [20] extracted the fundamental frequency of a bridge from the responses of an ordinary vehicle with its parameters calibrated in advance. Ubertini et al. [21] provided an estimate of the modal parameters of a newly built suspension with uncertainty bounds considering variations in identified modal features. Kong et al. [22] proposed an efficient method for numerical extraction of bridge modal properties from dynamic response of moving vehicles using a specialized test vehicle that consists of a tractor and two following trailers. Qin et al. [23] applied a kriging model and particle swarm optimization algorithm for the dynamic model updating of bridge structures using the higher vibration modes in a large-amplitude initial condition. Magalhaes et al. [24] presented an automated identification method of the modal parameters with the related bridge response under different wind conditions. Brownjohn [25] presented the first full modal survey of Jiangyin Yangtze River Bridge to identify the important features of the modal behavior. Guo et al. [26] presented a damage detection method based on the modal strain energy equivalence index (MSEEI) to solve structural multi-damage identification problems. Cui et al. [27] presented a damage detection method that was based on strain modes for beam-type structures with ambient excitation. Hou et al. [28] carried out the local structural damage Identification using Frequency-Domain Substructure Isolation Method. Liang et al. [29] performed damage detection of a real cable-stayed bridge using the proposed frequency co-integration technique, which could effectively eliminate the influence of the changing environmental temperature and accurately identify the structural damage. Li et al. [30] proposed a substructure damage identification method based on dynamic response reconstruction in the frequency domain, which was validated by a laboratory experiment of a steel plane frame structure. Zhang et al. [31] used the Substructure Virtual Distortion Method to rapidly construct the structural frequency response function for substructural damage identification of a frame structure based on structural modes.

Many studies of damage identification based on structural modal parameters have been carried out. However, practical difficulties exist in accurate damage identification due to the limited number of measurement points. Usually low-order modes are obtained, which are insensitive to local damage, especially for bridges that are large and complex. To increase the quantity of valid measured modal data, physical parameters are added to structures. Nalitolela et al. [32,33] proposed the mass and stiffness addition technique to perform structural modal updating, where modal information of the perturbed structures was utilized. Cha et al. [34] developed an approach to modal updating that involves the addition of known masses to the involved structure, where the modes of the modified system are employed in conjunction with the initial modal survey. Dems et al. [35] studied damage identification using modal, static and thermographic analysis by introducing additional control parameters, such as supports, mass or loads to increase the sensitivity with respect to the identified parameters. Dinh et al. [36] performed modal identification in several mass-modified conditions and verified the robustness of the identification numerically and experimentally. Lu et al. [37] analyzed the influence of the additional mass on the damage identification of a beam structure to improve the identification accuracy and discovered that the size, number, and position of the additional mass impact damage identification. Compared with other physical parameters, adding masses to physical structures can be easily performed. However, heavy structures such as bridges require a substantial amount of additional mass to enable distinct changes in the structural dynamic characteristics and improve their sensitivity to damage, which may cause structural overloading or damage. Zhang et al. [38,39] introduced a virtual control system to improve the accuracy of identification. Hou et al. [40] proposed a damage identification method in which virtual masses are added to the original structure based on the Virtual Distortion Method [41], where the frequency responses of the virtual structure with additional virtual mass can be constructed using the measured excitation and the related acceleration responses of the original structure. This method can afford a sufficient amount of modal information with a high sensitivity to local damage. Hou et al. [42] extended the approach based on virtual masses with Bayesian theory and verified it by experiments with a 3-story frame structure.

This paper presents a damage identification method for bridges that utilizes additional virtual mass. Traditional methods of exciting a bridge using a hammer or a shaker have the limitations of small impact energy [43]. A vehicle bump is chosen in this paper to excite the responses required for constructing a virtual structure with an additional virtual mass. The bump test is easily performed in practice. However, vehicle action can modify the dynamic characteristics of the bridge, and the coupling between the bridge and the vehicle should be considered. Tan et al. [44,45,46] divided the process of the vehicle bump into three stages: the static state before the bump, the bump itself, and the free-decay vibration after the bump. They computed the dynamic response in each stage, and the computed strain responses were similar to those of the field test. In references [44,45,46], the static strain state was analyzed before the bump because the static force also resulted in a displacement. In this paper, the acceleration of the bridge is required for the application of the additional virtual mass method, and therefore, the bridge state before the bump does not have to be calculated since no acceleration occurs when the vehicle is stationary. The structural acceleration response generally contains a larger amount of high-frequency information about the bridge. A detailed analysis of a bump-induced excitation is presented in four stages: separation, free-fall, contact, and coupled vibrations, in order to accurately simulate the structural acceleration responses with a vehicle bump.

The paper is structured as follows: first, general formulas are derived for the response of a virtual structure with additional virtual parameters, including virtual mass, stiffness, and damping. Second, the method of damage identification based on additional virtual mass is reformulated. Third, vehicle bump-induced excitation is analyzed in four stages. Fourth, the main steps of the proposed methodology are stated. Last, a numerical example of a two-span continuous beam is used to verify the proposed method using a 1-degree of freedom (DOF) car model and 4-DOF car model.

## 2. Damage Identification Based on Additional Virtual Masses

First, a general formula for constructing the responses of a virtual structure with additional virtual physical parameters, such as mass, damping and stiffness, is introduced. Second, the calculation formula related to only additional virtual mass is provided. Last, an objective function for damage identification based on the additional virtual mass is presented.

### 2.1. Virtual Structure with Additional Virtual Physical Parameters

The structural modal parameters are inherent characteristics of a structure. The structural damage can be determined by comparing the modal parameters of the intact structure and the damaged structure. Assume a structure with *n_d_* DOFs. The mass matrix, damping matrix and stiffness matrix of the original intact structure are denoted as *M*, *C* and *K*, respectively. Thus, the equation of motion in the frequency domain is as follows:

(1) $$M\ddot{X}\left(\omega \right)\;+\;C\dot{X}\left(\omega \right)\;+\;KX\left(\omega \right)\;=\;BF\left(\omega \right)$$

where F(ω) is the excitation vector composed of *n_f_* excitations applied to the structure and *B* represents the excitation position matrix. The response is expressed in Equation (2), as follows:

(2) Y(ω) = H(ω)BF(ω)

where Y(ω) is the structural response measured by involved sensors for the given excitation; and H(ω) is the related frequency response function (FRF) of the structure. 

Assume that the changes to the mass, damping and stiffness of the original structure occur in the form of the incremental matrices ΔM,ΔC,ΔK, respectively. The equation of motion of the modified structure can be expressed in the frequency domain as:

(3) $$\left(M\;+\;\Delta M){\ddot{X}}^{V}\left(\omega \right)\;+\;\left(C\;+\;\Delta C\right){\dot{X}}^{V}\left(\omega \right)\;+\;\left(K\;+\;\Delta K\right)X^{V}\left(\omega \right)\;=\;BF(\omega \right)$$

where $X^{V}\left(\omega \right)$ refers to the responses of the modified structure. By moving the incremental terms to the right side of Equation (3), it can be reformulated as Equation (4):

(4) $$M{\ddot{X}}^{V}\left(\omega \right)\;+\;C{\dot{X}}^{V}\left(\omega \right)\;+\;KX^{V}\left(\omega \right)\;=\;BF\left(\omega \right)\;-\;\Delta M{\ddot{X}}^{V}\left(\omega \right)\;-\;\Delta C{\dot{X}}^{V}\left(\omega \right)\;-\;\Delta KX^{V}\left(\omega \right)$$

In Equation (4), the incremental matrices ΔM,ΔC,ΔK that represent the related modifications can be expressed as $\Delta M\;=\;T_{m}^{T}\Delta_{m}T_{m}$, $\Delta C\;=\;T_{c}^{T}\Delta_{c}T_{c}$, $\Delta K\;=\;T_{k}^{T}\Delta_{k}T_{k}$, respectively, where $T_{m}^{T},T_{c}^{T},T_{k}^{T}$ represents the coordinate transformation matrices for structural mass, damping and stiffness. The parameters $\Delta_{m},\;\Delta_{c},\Delta_{k}$ represent the mass, damping and stiffness modification values, respectively. Let ${\ddot{z}}^{V}\;=\;T_{m}{\ddot{X}}^{V}$*,* ${\dot{z}}^{V}\;=\;T_{c}{\dot{X}}^{V}$, $z^{V}\;=\;T_{k}X^{V}$, and $Z^{V}\;=\;{\left[{\ddot{z}}^{V}(\omega )\;{\dot{z}}^{V}(\omega )\;z^{V}(\omega )\right]}^{T}$, and substitute them into Equation (4) to obtain the response of the modified structure, expressed as follows:

(5) $$\begin{array}{ll} Y^{V}\left(\omega \right)\;=\;H\left(\omega \right) & \left[BF\left(\omega \right)\;-\;T_{m}^{T}\Delta_{m}{\ddot{z}}^{V}\;-\;T_{c}^{T}\Delta_{c}{\dot{z}}^{V}\;-\;T_{k}^{T}\Delta_{k}z^{V}\right]\\ & =\;Y\left(\omega \right)\;-\;\left[\begin{array}{ccc} H\left(\omega \right)T_{m}^{T}\Delta_{m} & H\left(\omega \right)T_{c}^{T}\Delta_{c} & H\left(\omega \right)T_{k}^{T}\Delta_{k} \end{array}\right]Z^{V} \end{array}$$

Let $h_{m}\left(\omega \right)\;=\;H\left(\omega \right)T_{m}^{T}$, $h_{c}\left(\omega \right)\;=\;H\left(\omega \right)T_{c}^{T}$, and $h_{k}\left(\omega \right)\;=\;H\left(\omega \right)T_{k}^{T}$, and assemble all matrices into a matrix $h_{v}\;=\;[\begin{array}{ccc} h_{m} & h_{c} & h_{k} \end{array}]$. In this way, Equation (5) can be further simplified into Equation (6) as:

(6) $$Y^{V}\left(\omega ,\Delta \right)\;=\;Y\left(\omega \right)\;-\;h_{v}\Delta_{v}Z^{V}$$

where the diagonal matrix $\Delta_{v}\;=\;\mathrm{Diag}\;\left[\begin{array}{ccc} \Delta_{m} & \Delta_{c} & \Delta_{k} \end{array}\right]$. Let P be the observation matrix that is an invertible matrix related to the sensors adopted in the test. The responses of the modified structure $Y_{}^{V}$ can be expressed as follows:

(7) $$Y^{V}\;=\;PZ^{V}$$

To construct the responses of the modified structure using the measured responses of the actual structure, sensors are placed in positions where the structure has changed locally, and the excitations are applied at the same locations as the sensors. Let $Q_{ij}\left(\omega \right)$ and ${\bar{Y}}_{ij}\left(\omega \right)$ represent the excitation and response, respectively, at the *i*th position, where the additional parameters are applied to the *j*-th group test. Denote $Q\left(\omega \right)\;=\;\left[Q_{ij}\left(\omega \right)\right]$ and $\bar{Y}\left(\omega \right)\;=\;\left[{\bar{Y}}_{ij}\left(\omega \right)\right]$. The excitation and the measured responses of the actual structure $\bar{Y}\left(\omega \right)$ can be related as in Equation (8),

(8) $$\bar{Y}\left(\omega \right)\;=\;h\left(\omega \right)Q\left(\omega \right)$$

By combining Equations (6)–(8), the frequency response $H_{\Delta }^{v}\left(\omega ,\Delta_{v}\right)$ of the modified structure with modified or additional physical parameters $\Delta_{v}$ can be expressed as in Equation (9):

(9) $$H_{\Delta }^{v}\left(\omega ,\Delta_{v}\right)\;=\;{\left(I\;+\;\bar{Y}\left(\omega \right)Q{\left(\omega \right)}^{-1}\Delta_{v}P^{-1}\right)}^{-1}\bar{Y}(\omega )Q{\left(\omega \right)}^{-1}$$

In Equation (9), the matrix Q(ω) is a square matrix for computing its inverse, and the number of test groups should be equivalent to the number of additional virtual physical parameters.

For any system modification parameter $\Delta_{v}$, the responses of the modified structure can be calculated using Equation (9), which only requires the responses of the original structure $\bar{Y}\left(\omega \right)$ and the corresponding excitation Q(ω) to be measured or determined in advance. In this way, the responses of the structure with modified parameters can be numerically constructed without adding any real physical parameters to the actual structure. Such a modified structure is referred to as a virtual structure with additional virtual mass, damping and stiffness. In practice, the additional virtual physical parameters may be selected according to the specific project conditions, that is, $\Delta_{v}$ may consist of the additional mass $\Delta_{m}$, damping $\Delta_{m}$ or stiffness $\Delta_{k}$, or a combination of these parameters. Note that the adopted sensors should correspond to the type of the additional virtual parameters, that is, the acceleration sensor corresponds to the virtual mass, the velocity transducer corresponds to the virtual damping, and the displacement sensor corresponds to the virtual stiffness. 

### 2.2. Additional Virtual Mass Method

In practical engineering, the acceleration response of a structure and the acceleration that corresponds to the virtual mass are often measured. Thus, this paper adopts the additional virtual mass method to perform damage identification.

When multiple excitations are applied to a structure, a joint solution is required to obtain the responses of the virtual structure. Assume that the number of excitations is *n_f_*, then *n_f_* groups of dynamic tests are required to solve Equation (9) and each group of excitations must be different. The load time history of each excitation is measured to form the excitation matrix Q(ω). The acceleration sensors are arranged only in the positions of the excitations, and the corresponding responses are assembled to form the responses matrix $\bar{Y}(\omega )$. Equation (9) is rewritten as the frequency response $H_{\Delta }^{v}\left(\omega ,\Delta_{m}\right)$ of the virtual structure with the additional virtual mass $\Delta_{m}$:

(10) $$H_{\Delta }^{v}\left(\omega ,\Delta_{m}\right)\;=\;{\left(I\;+\;\bar{Y}(\omega )Q{\left(\omega \right)}^{-1}\Delta_{m}P^{-1}\right)}^{-1}\bar{Y}(\omega )Q{\left(\omega \right)}^{-1}$$

**Figure 1:**
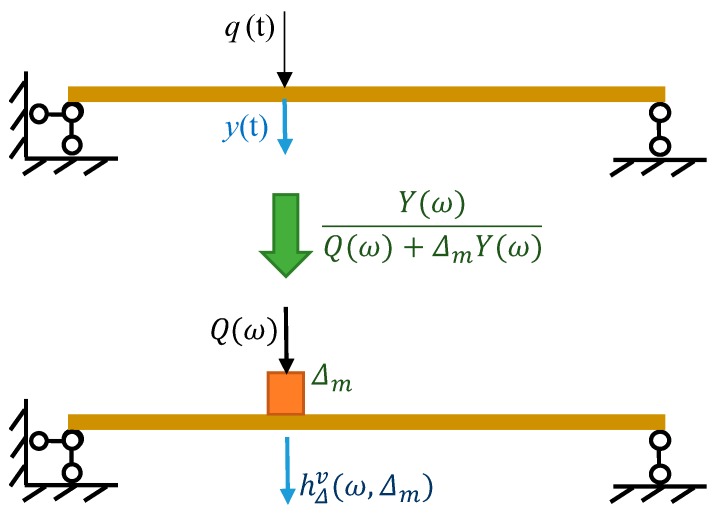
Diagram of the virtual mass construction.

When a single excitation is applied to the structure, only one sensor is required to measure the responses. Equation (10) can be further simplified. Figure 1 shows a diagram of the virtual mass construction using a single excitation. Denote by *y*(*t*) the structural acceleration measured in the same DOF as the single excitation *q*(*t*). Let Y(ω) and Q(ω) be the Fourier transforms of the acceleration and the excitation, respectively. 

For a virtual structure with an added virtual mass $\Delta_{m}$, its frequency response $h_{\Delta }^{v}\left(\omega ,m_{v}\right)$ in Figure 1 can be calculated using Equation (11), which is a simplified form of Equation (10), as deduced in detail in [42]:

(11) $$h_{\Delta }^{v}\left(\omega ,\Delta_{m}\right)\;=\;\frac{Y\left(\omega \right)}{Q\left(\omega \right)\;+\;\Delta_{m}Y\left(\omega \right)}$$

Using the constructed frequency response $H_{\Delta }^{v}\left(\omega ,\Delta_{m}\right)$ or $h_{\Delta }^{v}\left(\omega ,m_{v}\right)$, the modes of the virtual structure with the added virtual mass $\Delta_{m}$ can be identified as the indicators for damage identification.

### 2.3. Damage Identification

In practice, for complex structures such as bridges, it is generally feasible to divide them into substructures, and to identify the substructural damage. For each substructure, a virtual mass is applied, and then the natural frequencies of the corresponding virtual structures are identified using the structural frequency responses $h_{\Delta }^{v}\left(\omega ,m_{v}\right)$ constructed via Equation (11). Denote by $\omega_{ij}^{m}$ as the identified *i*-th frequency of the virtual structure with the virtual mass applied to the *j*-th substructure. All identified frequencies are combined to identify the structural damage via the following equation:

(12) $$\Delta \left(\mu \right)\;=\sum q_{ij}\left|\omega_{ij}^{A}\left(\mu \right)\;-\;\omega_{ij}^{m}\right|/\omega_{ij}^{m}$$

where $\omega_{ij}^{A}\left(\mu \right)$ is the calculated *i*-th frequency of the finite element model of the virtual structure with the virtual mass applied to the *j*-th substructure under the given damage μ, and $q_{ij}$ are the weight coefficients.

## 3. Process Analysis of Vehicle Bumps

Damage identification using additional virtual mass [42] requires that the excitation be known. A modal force hammer is often used to constitute the additional virtual mass, but it has a limited impact energy. In recent years, a vehicle bump has become popular as an excitation due to its convenient application in practice with an impact energy sufficiently large for a bridge structure. However, a bump-induced excitation is complex because the vibrations of the vehicle and the bridge are coupled. Therefore, a precise analysis of the entire process of the bump-induced excitation is necessary to provide an accurate excitation time history, as is required for the construction of the additional virtual mass. 

**Figure 2:**
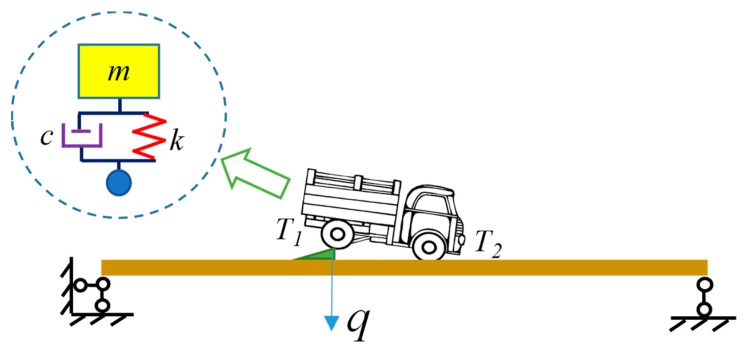
Diagram of vehicle bump on a bridge using a 1-DOF vehicle model.

**Figure 3:**
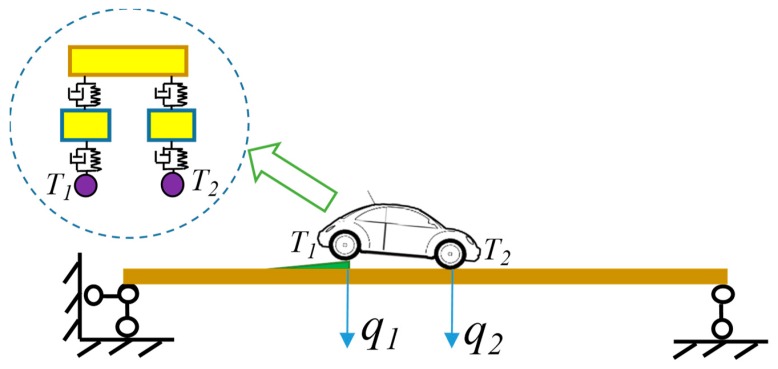
Diagram of vehicle bump on a bridge using a 4-DOF vehicle model.

In this paper, first, a 1-DOF vehicle model with is employed as an example to analyze the entire process of the bump-induced excitation, as shown in Figure 2. Second, a 4-DOF vehicle model is considered in the bump analysis, as shown in Figure 3, where one wheel is on the bump board, and the other wheel stops on the bridge. Denote the left wheel as the *T*_1_ wheel, and the right wheel as the *T*_2_ wheel. The analysis of the *T*_1_ wheel bump is the same as that of the 1-DOF vehicle model in Figure 2, with the exception that the coupled vibration of wheel *T*_2_ with the bridge is considered during the process. For simplicity, only a 1-DOF vehicle model is considered as an example to illustrate the analysis of the bump-induced excitation. In the numerical simulation, a more complex 4-DOF vehicle model is adopted. 

**Figure 4:**
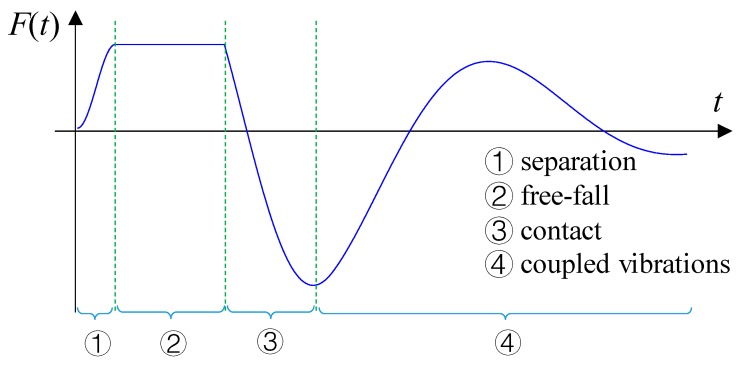
Four stages of bump excitation.

The vehicle is modeled as a single mass-spring, as shown in Figure 2. Let *m*, *c*, *k* denote respectively the mass, damping, and stiffness of the vehicle. The motion of the vehicle is a free-fall after leaving the jumping board, and when the vehicle contacts the bridge, the vehicle and bridge vibrate simultaneously. Generally, a bump-induced excitation was often analyzed as an impact applied to a structure. This paper presents a detailed analysis of the bump-induced excitation in four stages: separation, free-fall, contact and coupled vibrations, see Figure 4. 

### 3.1. Separation Stage

Before the bump test, the vehicle stops on a triangle board, and its weight *F_mg_* = *mg* causes a deformation of the bridge. The system state at this moment is defined as the equilibrium state. Due to the elastic deformation of the vehicle tire and its non-planar surface, the separation of the vehicle from the board requires the time *t*_1_, although it is very short. The pressure of the vehicle to the bridge gradually decreases from *F_mg_* to zero during the time period [0, *t*_1_], and the corresponding structural vibration can be found by integrating Equation (13):

(13) $$M\ddot{x}\left(t\right)\;+\;C\dot{x}\left(t\right)\;+\;Kx\left(t\right)\;=\;F\left(t\right)$$

(14) $$F\left(t\right)\;=\;mg\left(1-cos\left(t\pi /t_{1}\right)\right)/2,\;t\in \left[0,t_{1}\right]$$

**Figure 5:**
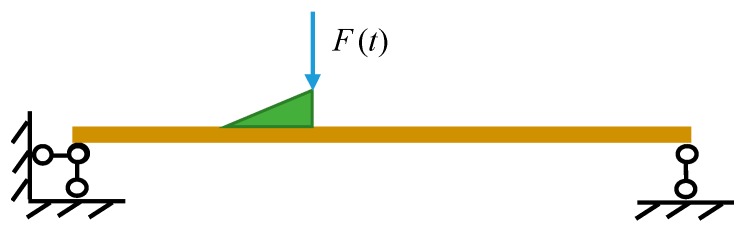
Force analysis in the separation period.

The decreasing vehicle pressure is approximatively simulated by the excitation *F*(*t*) using the cosine function shown in Equation (14), which is applied at the position of the vehicle, as shown in Figure 5:

### 3.2. Free-Fall Stage

In this stage, the vehicle has left the jumping board as a free-falling body. The height of the board is *h*. The time $t_{h}\;=\;\sqrt{2h/g}$ is required for the vehicle to fall to the bridge with the velocity *v*_0_ = *t_h_ g*. Denote the time moment *t*_2_ = *t*_1_ + *t_h_*. The bridge vibration can be analyzed using Equation (13), where the excitation *F*(*t*) is equal to the weight of the vehicle *mg*, that is:

*F*(*t*) = *mg*, *t*∈[*t*_1_, *t*_2_] (15)

### 3.3. Contact and Impact Stage

**Figure 6:**
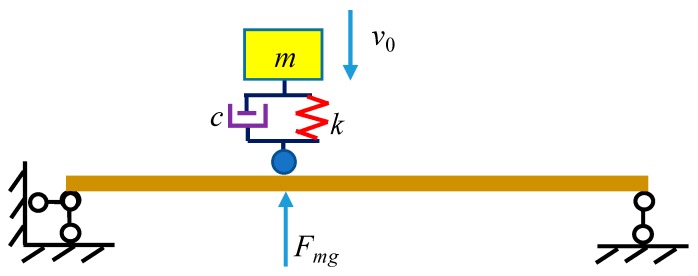
Interface between the vehicle and the bridge in the contact stage.

In this stage, as shown in Figure 6, the vehicle and bridge simultaneously vibrate with their initial states obtained from the last period. Denote by *v*_0_ the initial velocity of the vehicle at the contact moment, and let *x*_b_ be the initial state of the bridge. Traditionally, the vehicle tire force applied to the bridge at the initial contact is equal to *cv*_0_, where *c* is the vehicle damping, and the impact is large. Since the vehicle tire is constructed of rubber, the interface force is assumed to gradually increase. The damping of vehicle tire *c*_3_(*t*) is assumed to change with time as in Equation (16), where *t*_3_ is the moment of the largest deformation of the vehicle spring:

*c*_3_(*t*) = *c*(*t* − *t*_2_)/(*t*_3_ − *t*_2_), *t*∈[*t*_2_, *t*_3_] (16)

Trial and iteration are required to determine *t*_3_ and *c*_3_(*t*). The main steps are described as follows: first, given an initial large value of *t*_3_, *c*_3_(*t*) is determined via Equation (16). Second, the vibration of the vehicle in Figure 6 during time period [*t*_2_, *t*_3_] is computed by integrating Equation (17) with the initial velocity *v*_0_ and the initial state *x*_b_ of the bridge. Based on the results, the maximum deformation moment $t_{3}^{\prime }$ during time [*t*_2_, *t*_3_] for the next iteration can be determined. Set $t_{3}\;=\;t_{3}^{\prime }$, and then the steps are repeated until the values of $t_{3}^{\prime }$ in two consecutive iterations are similar:

(17) $$\tilde{M}{\ddot{x}}_{s}\left(t\right)\;+\;\tilde{C}{\dot{x}}_{s}\left(t\right)\;+\;\tilde{K}x_{s}\left(t\right)\;=\;F\left(t\right)$$

where $\tilde{M},\tilde{C},\;\tilde{K}$ are respectively the mass, damping and stiffness matrix of the coupled vehicle-bridge system, including the variable vehicle damping *c*_3_(*t*), the vehicle mass *m* and the vehicle stiffness *k*.

We denote by *x*_c_(*t*) and *v*_c_(*t*) the relative displacement and velocity between the vehicle and the bridge, which are computed using Equation (17). The vehicle tire force, i.e., the excitation applied to the bridge *F*(*t*), can be computed by the following Equation (18):

*F*(*t*) = *mg* − *c*_3_(*t*)*v*_c_(t) − *kx*_c_(*t*), *t*∈[*t*_2_, *t*_3_] (18)

### 3.4. Coupled Vibration

In this stage, the vibration response of the coupled system can be computed by Equation (17), where the vehicle damping is *c*, and the excitation applied to the bridge *F*(*t*) is expressed as:

*F*(*t*) = *mg* − *cv*_c_(t) − *kx*_c_(*t*), *t* > *t*_3_ (19)

## 4. Procedure of the Proposed Methodology

Based on the additional virtual mass method, the vehicle-bump is taken as an excitation to identify the bridge damage. The main steps are as follows:

(1) Locate a vehicle, acceleration sensors and force sensors on the bridge properly. The number of the sensors should correspond to the number of the excitations caused by the vehicle. For example, one acceleration sensor and one force sensor are required if 1-DOF vehicle model is adopted, while two acceleration sensors and two force sensors are demanded if a two-wheel vehicle model is used. In addition, sensors should be located along the DOF of the respective excitations.
(2) In the field test, both the bump-induced excitation and the structural accelerations are measured. In the numerical simulation of this paper, the bump-induced excitation and the accelerations are computed as described in Section 3.
(3) Perform the Fourier transform of the excitation and the measured accelerations respectively to obtain the frequency responses of the original structure, and then substitute the virtual additional mass value to construct the frequency responses of the virtual structure by Equation (10).
(4) Using the constructed frequencies and the finite element model, minimize the objective function Δ(μ) in Equation (12) for damage identification.

## 5. Numerical Verification

**Figure 7:**
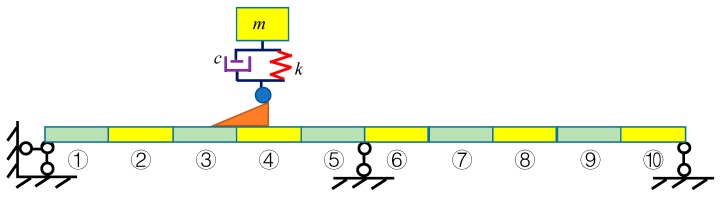
Two-span continuous bridge with a 1-DOF vehicle model.

The two-span continuous bridge model shown in Figure 7 is employed to verify the proposed methodology. The length of each span is 24 m. The bending stiffness *EI* is 1.2 × 10^9^ N/m^2^ and the mass is 1.0 × 10^4^ kg/m. The height of the jumping board is 0.1 m. The structure is divided into 10 substructures. In the finite element model, each substructure has two elements. Damage identification with a 1-DOF and 4-DOF vehicle models are discussed in this section.

### 5.1. Structural Damage Identification Using 1-DOF Vehicle Model

**Figure 8:**
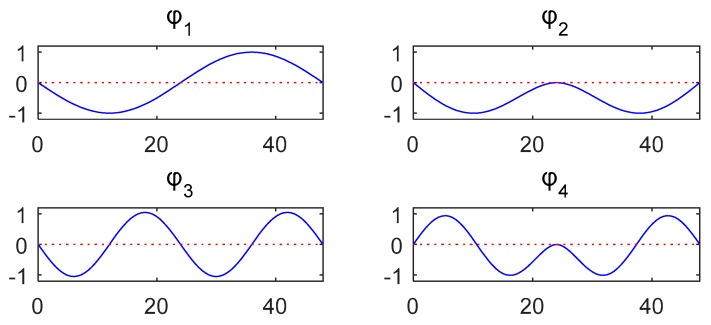
First four modal shapes of the bridge structure.

The vehicle mass is *m* = 2.0 × 10^3^ kg, the damping is *c* = 1.5 × 10^4^ kg/s, and the stiffness is *k* = 3.5 × 10^5^ N/m. The first four modal shapes and natural frequencies of the bridge are shown in Figure 8 and Table 1 “Bridge”. The vehicle is located in the middle of the fourth substructure shown in Figure 7 and simulated with a single-spring model. The first four natural frequencies of the coupled vehicle-bridge system are listed in Table 1 “With Spring”. If the vehicle is simulated as a point mass without the spring, the first four natural frequencies of the coupled system are shown in Table 1 “Only Mass”. 

Table 1 shows that the natural frequencies of the system are dependent on the vehicle model, that is the vehicle model influences the system modal characteristics. In this paper, only the vehicle tire forces are considered, which avoids the influence of vehicle models. In the following sections, the vehicle located in the middle of the fourth substructure is considered as an example to illustrate the additional virtual mass construction using the vehicle bump-induced excitation.

#### 5.1.1. Analysis of Vehicle Bump-Induced Excitation

**Figure 9:**
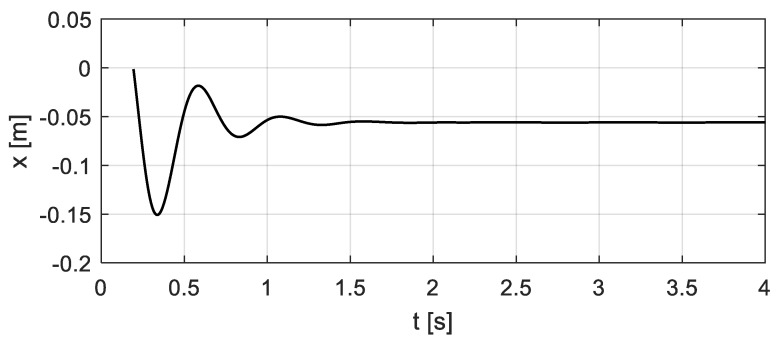
Relative displacement *x*_c_(*t*) between the vehicle and the bridge.

**Figure 10:**
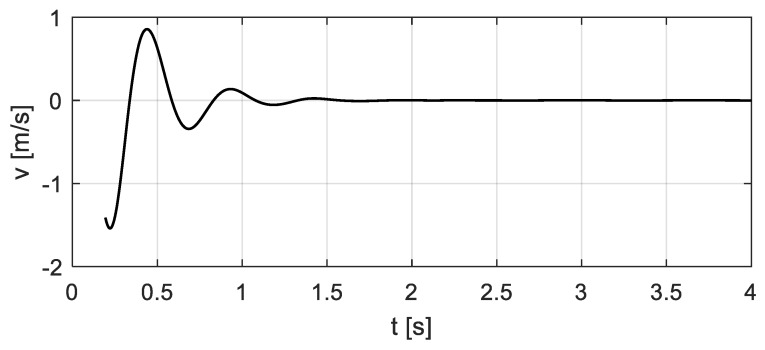
Relative velocity *v*_c_(*t*) between the vehicle and the bridge.

**Figure 11:**
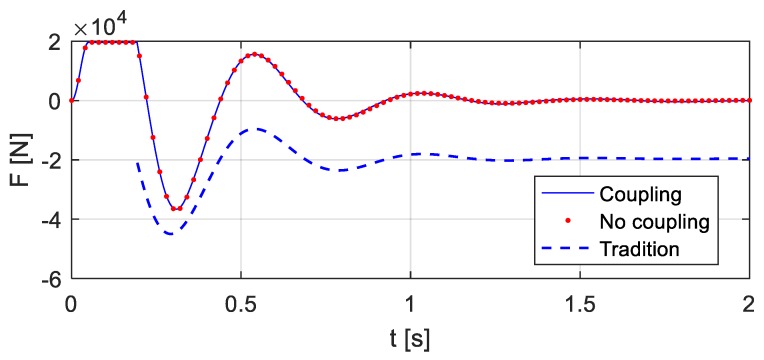
Comparison of the time history of vehicle bump-induced excitation considering different factors.

By the analysis in Section 3, considering the vehicle-bridge coupled system, the relative displacement *x*_c_(*t*) and velocity *v*_c_(*t*) between the vehicle and the bridge, as well as the entire time history of the vehicle bump-induced excitation *F*(*t*), can be computed, see Figure 9 and Figure 10. The corresponding excitation *F*(*t*) of the bridge is shown in Figure 11 ‘Coupling’.

Assume temporarily that the bridge is a rigid body, which means that the structural deformation is not considered. The corresponding bump-induced excitation is computed and shown in Figure 11 ‘No coupling’. Apparently, it seems to be similar to the excitation ‘Coupling’ in Figure 11. The bridge deformations caused by the bump-induced excitations are thus small. 

**Figure 12:**
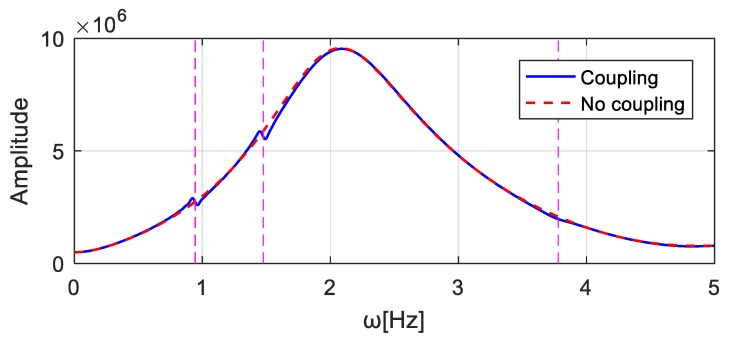
Comparison of the structural responses due to the bump-induced excitations in the frequency domain considering different factors.

However, if the frequency spectra of the responses in the ‘Coupling’ and ‘No coupling’ cases are computed and compared, see Figure 12, it turns out that there are clear differences in the crucial ranges near the bridge natural frequencies. Therefore, it is necessary to consider the bridge-vehicle coupled system.

**Figure 13:**
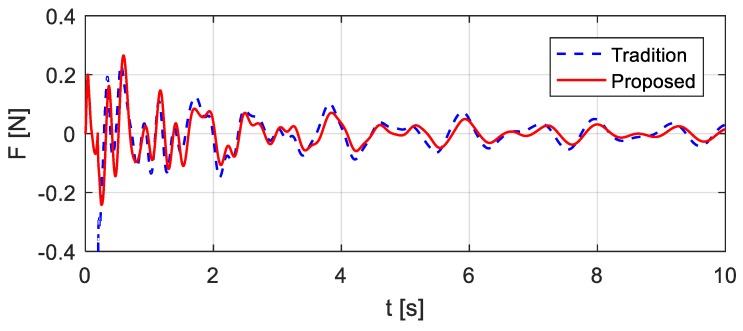
Comparison of the excitations determined by traditional studies and the proposed method.

In existing studies of bump excitations, a vehicle falls to the bridge with full contact, which means that the impact forces are instantaneously applied to the structure. In this way, the computed vehicle excitations are shown in Figure 11 ‘Tradition’, which are different from the excitations computed using the proposed method. The structural accelerations that are computed considering the instantaneous impact (Figure 13 ‘Tradition’) are compared with the accelerations that are computed using the proposed method (Figure 13 ‘proposed’), which shows that the differences primarily exist at the beginning of the structural responses. The structural free responses are concerned for the system modal identification in bridge monitoring, and thus the existing studies work well. However, the construction of the virtual additional mass in this paper requires the entire time history of the structural responses and demands a precise estimation of the responses. 

#### 5.1.2. Construction of Additional Virtual Mass

**Figure 14:**
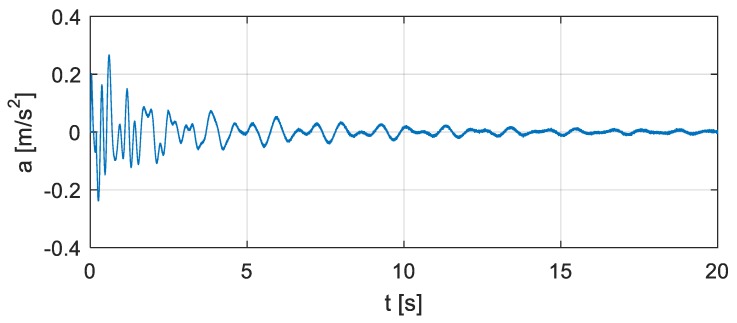
Vertical acceleration of the bridge at the location of the vehicle.

**Figure 15:**
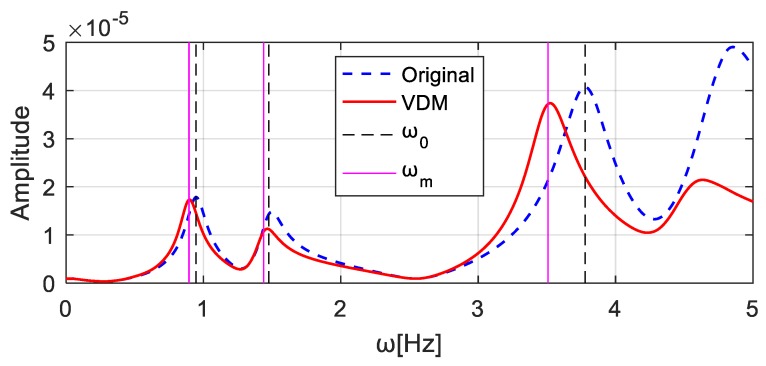
Frequency responses of the structure in different cases.

Figure 14 shows the vertical accelerations of the bridge at the location of the vehicle. They are computed for the coupled system (excitation ‘Coupling’ in Figure 11) and contain additionally 5% Gaussian random noise to simulate the measurement noise. The FFTs of the excitation and the acceleration are computed and used to determine the FRF of the original structure, as shown in Figure 15 ‘original’. Then a virtual additional mass, 20 times the actual vehicle mass, is employed to cause distinct changes in structural natural frequencies while avoiding an overload to the physical structure.

The value of the virtual additional mass, i.e., $\Delta_{m}$ = 4.0 × 10^4^ kg, is substituted into Equation (11). The frequency responses of the corresponding virtual structure can be constructed, see Figure 15 ‘VDM’. In Figure 15, the gridlines $\omega_{0}$ and $\omega_{m}$ denote respectively the natural frequencies of the original system and of the system with the additional virtual mass 4.0 × 10^4^ kg, as computed using the finite element model. The corresponding frequencies are listed in and denoted by “Original” and “With mass” in Table 2. The frequencies denoted as “VDM” in Table 2 refer to the natural frequencies of the virtual structure identified using the constructed frequency responses, as shown in Figure 15 ‘VDM’. They are very similar to the frequencies “With mass”, which confirms the effectiveness of the virtual additional mass method. 

#### 5.1.3. Damage Identification

**Figure 16:**
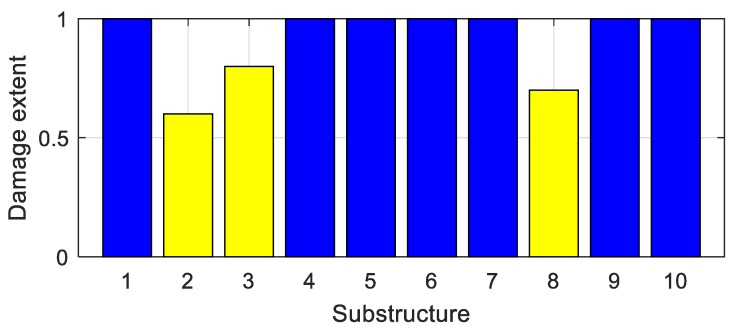
Damage extents of the substructures.

**Figure 17:**
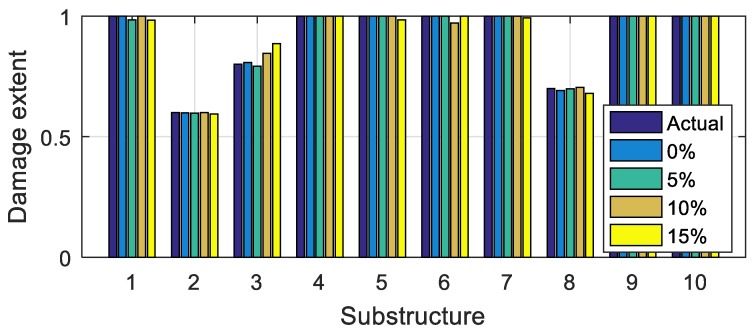
Damage identification results using 1-DOF vehicle model.

Assume that the 2nd, 3rd, and 8th substructure of the bridge is damaged with the damage extents shown in Figure 16. Vehicle bump tests are successively carried out in the middle of each substructure, and the corresponding bump-induced force is obtained via the analysis in Section 3. It is then applied to the middle of each substructure to construct the virtual structure with the large virtual additional mass $\Delta_{m}$ = 4.0 × 10^4^ kg. On each substructure, the vehicle bump tests is performed five times. Each time, the frequencies of the virtual structure are identified using the constructed responses (Equation (1)). The frequencies identified in the five tests are averaged to decrease the effects of the noise. In this way, for all ten substructures, ten groups of frequencies are obtained. Each group has three orders of frequencies; see Table 3 “VDM”. The frequencies that correspond to Table 3 “Actual” are computed using a finite element model of the actual damaged structure with an additional mass of 4.0 × 10^4^ kg, and they are found to be similar to the identified frequencies, which confirms the accuracy of the frequencies identified using the virtual additional mass method. Using the constructed frequencies and the finite element model, the objective function Δ(μ) in Equation (12) is built for damage identification, where $\omega_{ij}^{m}$ refers to the frequencies in Table 3 “VDM”, and $\omega_{ij}^{A}\left(\mu \right)$ are the values obtained using the finite element model. The optimization problem is solved using the function ‘Patternsearch’ from the optimization toolbox of MATLAB. Damage to the ten substructures is identified, as shown in Figure 17 and Table 4 (Noise 5%). The damage extents and locations can be accurately identified. 

To test the robustness of the proposed method to noise, damage identification is performed in the cases without noise and with 5%, 10% and 15% Gaussian random noise. The results are shown in Figure 17 and Table 4. The identification errors increase with the increasing noise level. The results are still acceptable in the case of 15% noise pollution, which results in the maximum error smaller than 10%. Therefore the proposed method is robust to noise. 

### 5.2. Structural Damage Identification Using 4-DOF Vehicle Model

**Figure 18:**
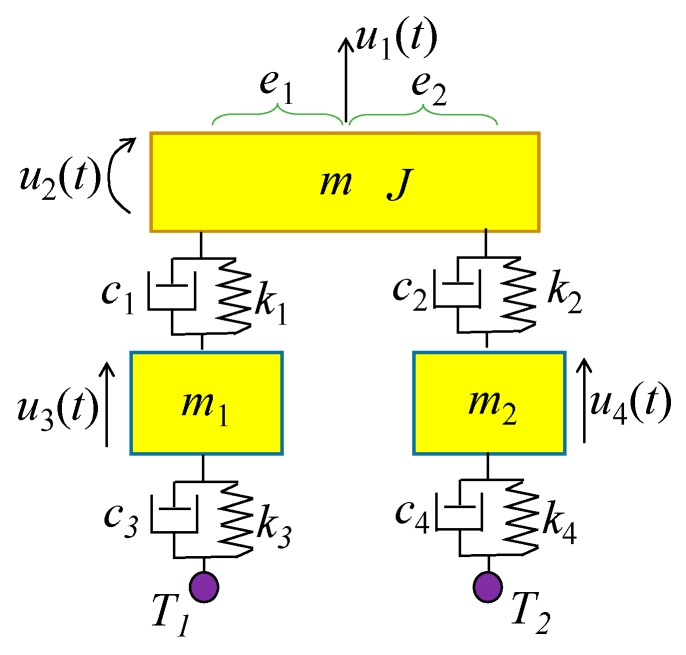
4-DOF car model.

A 4-DOF vehicle model is used to verify the effectiveness of the proposed method, as shown in Figure 18. Since the vehicle model has two wheels, both wheels generate excitation for the bridge, which is a multi-point excitation. Thus, simulation of two vehicle bumps are required to construct the responses of the virtual structure with the additional virtual mass, which can be calculated by Equation (10). Two wheels are adopted for the bump separately with the other wheel stopping on the bridge, and two virtual masses are added at the position of the two wheels in each test.

**Figure 19:**
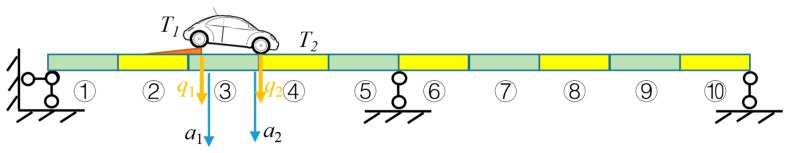
A two-span continuous bridge with 4-DOF vehicle model (*T*_1_ bump).

**Figure 20:**
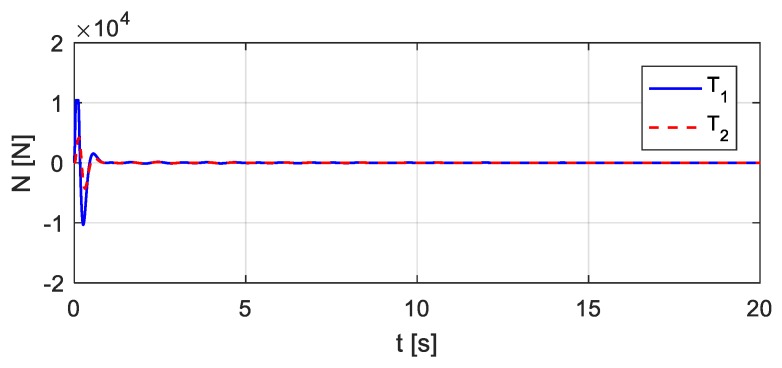
Estimated time histories of the excitations at wheels *T*_1_ and *T*_2_ in case wheel *T*_1_ bumps.

**Figure 21:**
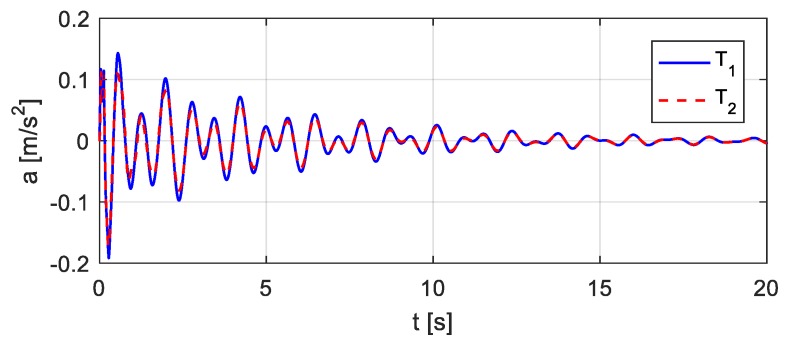
Estimated accelerations in case wheel *T*_1_ bumps.

**Figure 22:**
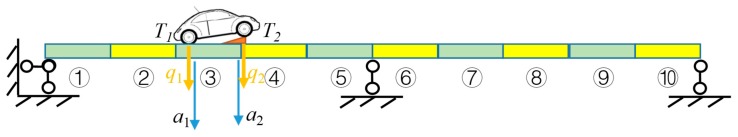
A two-span continuous bridge with 4-DOF vehicle model (*T*_2_ bump)).

**Figure 23:**
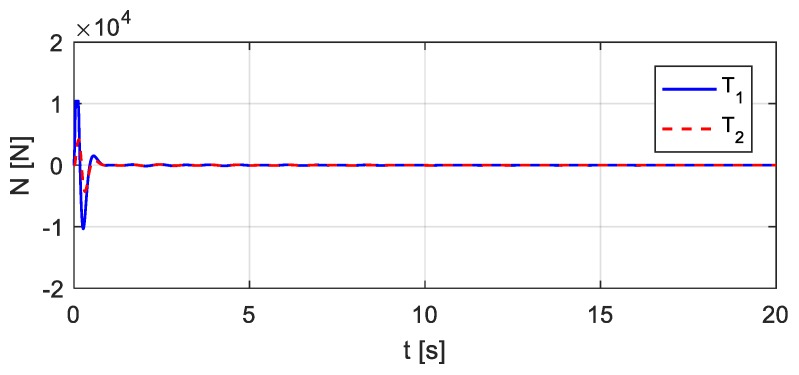
Estimated time histories of the excitations at wheel *T*_1_ and *T*_2_ in case wheel *T*_2_ bumps.

**Figure 24:**
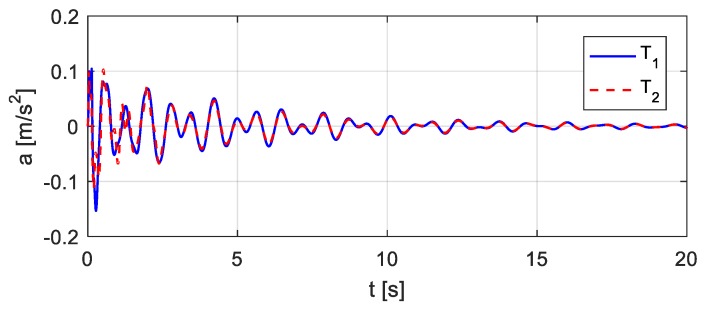
Estimated accelerations in case wheel *T*_2_ bumps.

The vehicle parameters assumed for Figure 18 are as follows: *m* = 1.75 × 10^3^ kg, *J* = 1.4 × 10^3^ kg∙m^2^, *m*_1_ = 1.0 × 10^2^ kg, *m*_2_ = 1.5 × 10^2^ kg, *k*_1_ = 4.0 × 10^5^ N/m, *k*_2_ = 3.0 × 10^5^ N/m, *k*_3_ = 1.0 × 10^5^ N/m, *k*_4_ = 1.0 × 10^5^ N/m, *c*_1_ = 1.5 × 10^4^ kg/s, *c*_2_ = 2.0 × 10^4^ kg/s, *c*_3_ = 1.5 × 10^4^ kg/s, *c*_4_ = 2.0 × 10^4^ kg/s, *e*_1_ = 1.35 m, *e*_2_ = 1.65 m. Let wheel *T*_1_ be located 10.5 m from the left end of the bridge, as shown in Figure 19. Consider that wheel *T*_1_ performs the bump and that the entire bump-induced excitation is analyzed as in Section 3, but the coupled vibration of the other wheel *T*_2_ with the bridge is considered during the process. The estimated time histories of the excitations at the wheels *T*_1_ and *T*_2_ are shown in Figure 20, and the corresponding acceleration responses are shown in Figure 21. Consider then wheel *T*_2_ to perform the bump, as shown in Figure 22. The estimated time histories of the excitations at wheel *T*_1_ and *T*_2_ are shown in Figure 23, and the corresponding acceleration responses are shown in Figure 24.

**Figure 25:**
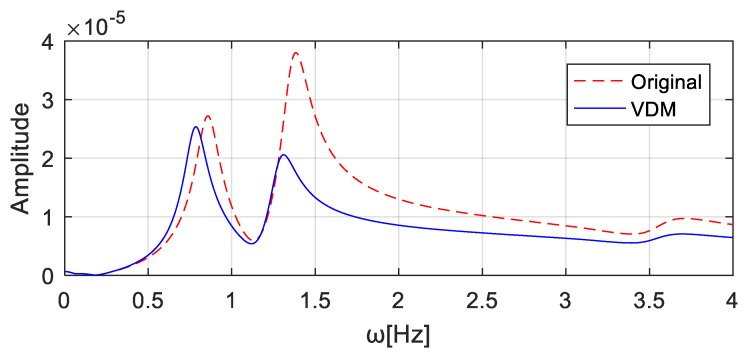
Acceleration frequency responses at *T*_1_ wheel position with the virtual mass added to the wheel *T*_1_.

**Figure 26:**
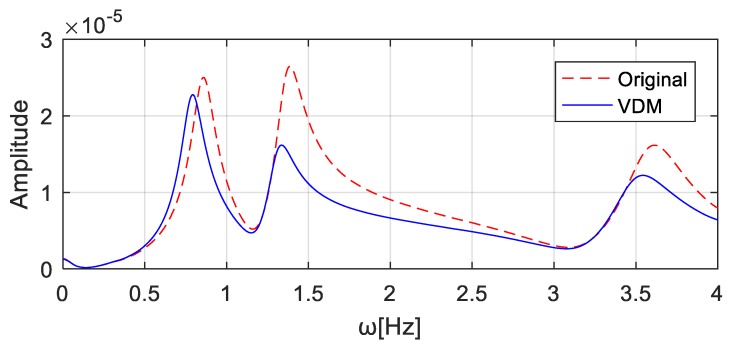
Acceleration frequency responses at *T*_2_ wheel position with the virtual mass added to the wheel *T*_2_.

To simulate noise pollution, 5% Gaussian white noise is added to the excitations and responses. If the virtual mass is 4.0 × 10^4^ kg at wheel *T*_1_ and 0 kg at wheel *T*_2_, the virtual mass matrix can be expressed as $\Delta_{m}\;=\;\left[\begin{array}{cc} 4 & 0\\ 0 & 0 \end{array}\right]\;\times 10^{4}$. The frequency response of the virtual structure is then obtained by substituting $\Delta_{m}$ and the Fourier transforms of the excitation and response into Equation (10). The computed acceleration frequency responses at the *T*_1_ wheel position are shown in Figure 25 ‘VDM’, and the first three natural frequencies of the structure can be identified as [0.7784, 1.2793, 3.5613] Hz. Similarly, the addition of the virtual mass of 4.0 × 10^4^ kg and 0 kg at position *T*_2_ and *T*_1_, respectively, results in the virtual mass matrix $\Delta_{m}\;=\;\left[\begin{array}{cc} 0 & 0\\ 0 & 4 \end{array}\right]\;\times \;10^{4}$. The computed acceleration frequency responses at the *T*_2_ wheel position are shown in Figure 26, and the identified first three natural frequencies of the virtual structure are [0.78584, 1.30304, 3.47824] Hz.

**Figure 27:**
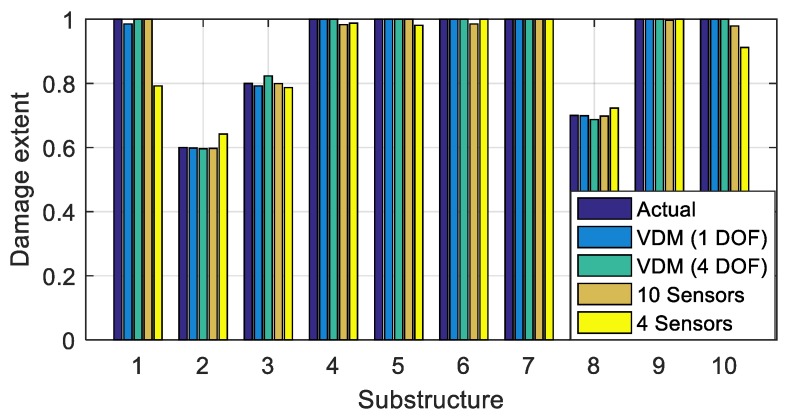
Comparison of damage identification results.

The wheel *T*_1_ is separately located at the positions [2.00 10.50 19.00 26.00 34.50 43.00] m on the bridge from its left end. At each position, two bumps are analyzed as described above, one for each wheel, and the corresponding excitations are computed. An additional virtual mass of 4.0 × 10^4^ kg is placed at each position, and the related responses and natural frequencies of the virtual structure are determined and listed in Table 5 “VDM”. In addition, the natural frequencies computed using the finite element model of the structure with the added masses (Table 5 “Actual”) are computed and found to be very similar to the VDM-estimated frequencies. The results confirm the accuracy of the estimated frequencies of the virtual structure using the constructed acceleration frequency responses via Equation (10). The damage to the bridge is identified using the frequencies in Table 5 “VDM” and shown in Figure 27 as “VDM (4 DOF)”. Both the locations and extents of damages can be accurately identified. The accuracy is almost the same as in the case of a 1-DOF vehicle, see also Table 6 (Error_1DOF, Error_4DOF).

### 5.3. Direct Identification of Damage Using Structural Modal Parameters

**Figure 28:**
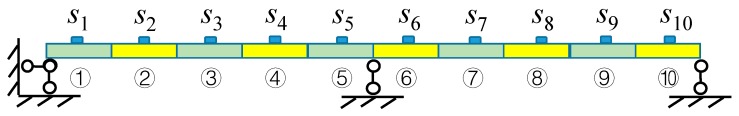
Location of the acceleration sensors.

**Figure 29:**
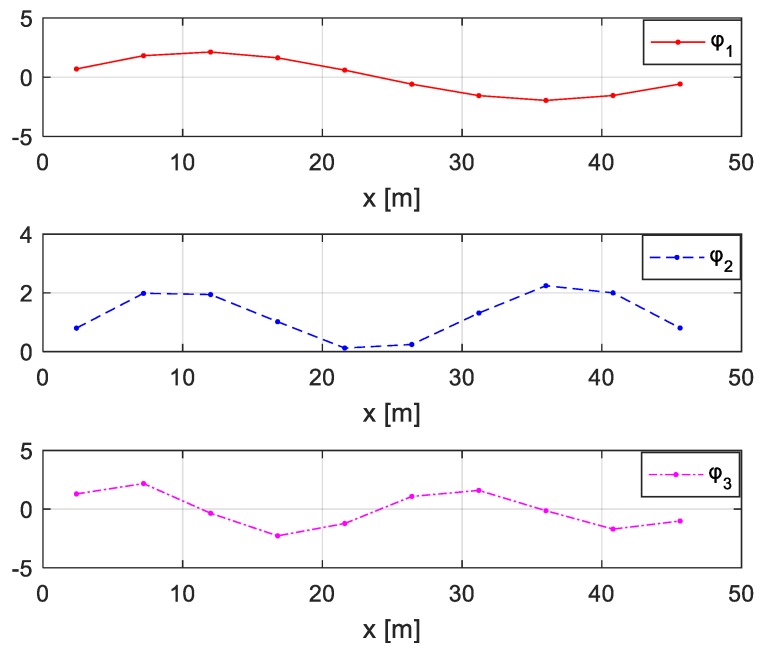
The identified first three modal shapes of the damaged bridge.

For comparison and in order to verify the advantage of the proposed method, the damages of the considered two-span bridge are also identified directly using structural modal parameters. The free vibration responses under environmental excitation are measured using acceleration sensors located in the middle of the ten substructures, as shown in Figure 28. Gaussian noise at the level of 5% is considered. First, the first three natural frequencies and mode shapes (Figure 29) are identified from the responses. The damages are optimized using the objective function considered in [47] and shown in Equation (20), where $n_{\varphi }$ is the number of the structural mode shapes and $n_{\omega }$ is the number of the natural frequencies, while $\alpha_{\varphi }$ and $\alpha_{\omega }$ are the respective weighting coefficients:

(20) $$f\left(\mu \right)\;=\;\alpha_{\varphi }\overset{n_{\varphi }}{\underset{i\;=\;1}{\sum }}\left(1\;-\;MAC\left(\varphi_{i}\left(\mu \right),{\bar{\varphi }}_{i}\right)\right)\;+\;\alpha_{\omega }\overset{n_{\omega }}{\underset{i\;=\;1}{\sum }}{\left(\frac{\omega_{i}\left(\mu \right)\;-\;{\bar{\omega }}_{i}}{{\bar{\omega }}_{i}}\right)}^{2}$$

(21) $$MAC\left(\varphi_{i}\left(\mu \right),{\bar{\varphi }}_{i}\right)\;=\;\frac{\left({\bar{\varphi }}_{i}^{T}\varphi_{i}\left(\mu \right)\right)\left(\varphi_{i}^{T}\left(\mu \right){\bar{\varphi }}_{i}\right)}{\left(\varphi_{i}^{T}\left(\mu \right)\varphi_{i}\left(\mu \right)\right)\left({\bar{\varphi }}_{i}^{T}{\bar{\varphi }}_{i}\right)}$$

First, all ten sensors S_1_~S_10_ are adopted, which yielded results similarly accurate as these of the proposed methodology, see Figure 27 “10 Sensors” and Table 6 (Dir_Ten, Error_ Ten). Then the damages are identified by employing four sensors S_2_, S_4_, S_7_, S_9_, located in the middle of the corresponding substructures as shown in Figure 28. The identification results are shown in Figure 27 “4 Sensors” and in Table 6 (Dir_Four, Error_ Four). The maximum error is large, i.e., over 20%.

### 5.4. Discussion of Results

In the numerical simulation, 1-DOF vehicle model is used to clearly introduce the method proposed in this paper. The bump-induced excitations computed using the 4-DOF model, shown in Figure 23, reveal that the excitation amplitude of the other wheel is as high as 30% of that of the bumping wheel. Therefore, in practice it is necessary to consider the 4-DOF model.

Then, using the computed bump-induced excitation, an additional virtual mass, 20 times the actual vehicle mass, is employed to increase the amount of the dynamic information gained from the tests. The natural frequencies of the virtual structure are identified, and the objective function is established for identification. The damage can be identified accurately using the vehicle bump and the additional virtual masses, even under relatively high levels of noise pollution, shown in Figure 17 and Figure 27, as well as in Table 4 and Table 6.

The proposed methodology, based on the vehicle bump and an additional virtual mass, requires only two acceleration sensors and two force sensors. For comparison, the tested typical identification method that used directly the modal parameters needed considerably more sensors to give similarly precise results. Such a low number of required sensors significantly facilitates practical implementation of the proposed approach. 

## 6. Conclusions

A damage identification method for bridge structures, based on vehicle bump-induced excitations and an additional virtual mass, is proposed. A numerical model of a two-span continuous beam is used to verify the effectiveness of the proposed method. The main conclusions can be stated as follows:

(1) The paper derives the basic equations used for constructing the frequency response of the virtual structure with additional virtual parameters (virtual mass, damping, and stiffness), which is a convenient and flexible way for effective addition of multiple physical parameters to the structure. The proposed approach broadens the potential application scope in practical engineering by using additional virtual physical parameters.
(2) The analysis of the vehicle bump is performed in four stages: separation, free-fall, contact, and coupled vibrations. Such an analysis yields accurate time histories of vehicle-induced excitations, which allows the additional virtual mass method to be applied to identify structural damage. The analysis can provide supportive evidence for analyzing bump-induced excitations in practice.
(3) Using the vehicle bump and the corresponding responses of the bridge, the responses of a virtual structure with an additional virtual mass can be accurately constructed to increase the response sensitivity to local damage. This allows the damage to be accurately identified with the error less than 10% even under 15% Gaussian noise pollution. The proposed process is effectively equivalent to an addition of a significant mass to the bridge for testing purposes, however without the risks of overloading the structure.
(4) A field validation of the proposed method is currently under preparation. The numerical computations of the bump-induced excitations will guide the height design of the springboard.

## Figures and Tables

**Table 1:** First four natural frequencies (Hz).

Order	First Order	Second Order	Third Order	Fourth Order
Bridge	0.945	1.476	3.779	4.783
With Spring	0.941	1.472	2.112	3.786
Only Mass	0.942	1.474	3.765	4.762

**Table 2:** First three natural frequencies (Hz).

	First Order	Second Order	Third Order
Original	0.945	1.476	3.779
With mass	0.895	1.439	3.509
VDM	0.896	1.438	3.517

**Table 3:** First four natural frequencies (Hz).

Vehicle Location	VDM			Actual		
	First Order	Second Order	Third Order	First Order	Second Order	Third Order
1	0.846	1.345	3.446	0.847	1.346	3.451
2	0.798	1.280	3.321	0.798	1.281	3.326
3	0.780	1.289	—	0.781	1.290	—
4	0.812	1.337	3.250	0.811	1.338	3.255
5	0.848	1.359	3.468	0.849	1.362	3.468
6	0.848	1.360	3.483	0.849	1.361	3.489
7	0.815	1.322	3.383	0.814	1.322	3.384
8	0.789	1.267	—	0.790	1.267	—
9	0.814	1.275	3.390	0.813	1.276	3.395
10	0.849	1.345	3.495	0.849	1.346	3.493

Note: ‘—’ denotes that the respective frequency is unattainable.

**Table 4:** Damage identification results using 1-DOF vehicle model under different noise levels.

Substructure	Actual	Noise Free	Noise 5%	Noise 10%	Noise 15%	Error_5% (%)	Error_10% (%)	Error_15% (%)
1	1	1	0.985	1	0.983	1.5	0	1.7
2	0.6	0.599	0.598	0.600	0.594	0.2	0	0.6
3	0.8	0.808	0.792	0.846	0.886	0.8	4.6	8.6
4	1	1	1	1	1	0	0	0
5	1	1	1	1	0.985	0	0	1.5
6	1	1	1	0.971	1	0	2.9	0
7	1	1	1	1	0.993	0	0	0.7
8	0.7	0.691	0.699	0.704	0.68	0.1	0.4	2
9	1	1	1	1	1	0	0	0
10	1	1	1	1	1	0	0	0

**Table 5:** First four natural frequencies (Hz).

Vehicle Location (m)	Mass Location	VDM			Actual		
		First Order	Second Order	Third Order	First Order	Second Order	Third Order
2.00	*T*_1_	0.849	1.350	3.489	0.849	1.350	3.486
	*T*_2_	0.822	1.304	3.273	0.822	1.305	3.271
10.50	*T*_1_	0.778	1.279	3.561	0.780	1.281	3.556
	*T*_2_	0.786	1.303	3.478	0.786	1.304	3.484
19.00	*T*_1_	0.830	1.354	3.276	0.831	1.355	3.270
	*T*_2_	0.851	1.348	3.495	0.851	1.362	3.499
26.00	*T*_1_	0.851	1.360	3.511	0.851	1.362	3.513
	*T*_2_	0.832	1.346	3.360	0.832	1.347	3.359
34.50	*T*_1_	0.793	1.279	3.550	0.793	1.280	3.556
	*T*_2_	0.792	1.261	3.547	0.792	1.261	3.540
43.00	*T*_1_	0.832	1.305	3.358	0.832	1.305	3.362
	*T*_2_	0.851	1.350	3.515	0.851	1.351	3.517

**Table 6:** Damage identification results in different cases with 5% noise.

Substructure	Actual	VDM- 1DOF	VDM- 4DOF	Dir -Ten	Dir -Four	Error_1DOF (%)	Error_4DOF (%)	Error_Ten (%)	Error_Four (%)
1	1	0.985	1	1	0.792	1.5	0	0	20.8
2	0.6	0.598	0.596	0.597	0.642	0.2	0.4	0.3	4.2
3	0.8	0.792	0.823	0.799	0.787	0.8	2.3	0.1	1.3
4	1	1	1	0.983	0.988	0	0	1.7	1.2
5	1	1	1	1	0.981	0	0	0	1.9
6	1	1	1	0.985	1.000	0	0	1.5	0
7	1	1	1	1	1.000	0	0	0	0
8	0.7	0.691	0.687	0.698	0.723	0.9	1.3	0.2	2.3
9	1	1	1	0.997	1.000	0	0	0.3	0
10	1	1	1	0.979	0.912	0	0	2.1	8.8
